# Pleomorphic Adenoma Gene 1 Is Needed For Timely Zygotic Genome Activation and Early Embryo Development

**DOI:** 10.1038/s41598-019-44882-0

**Published:** 2019-06-10

**Authors:** Elo Madissoon, Anastasios Damdimopoulos, Shintaro Katayama, Kaarel Krjutškov, Elisabet Einarsdottir, Katariina Mamia, Bert De Groef, Outi Hovatta, Juha Kere, Pauliina Damdimopoulou

**Affiliations:** 1 Department of Biosciences and Nutrition, Karolinska Institutet, SE-14186 Stockholm, Sweden; 2 Bioinformatics and Expression Analysis core facility, Department of Biosciences and Nutrition, Karolinska Institutet, SE-14186 Stockholm, Sweden; 3 Competence Centre on Health Technologies, 50410 Tartu, Estonia; 4 Molecular Neurology Research Program, University of Helsinki and Folkhälsan Institute of Genetics, 00014 Helsinki, Finland; 5 Department of Clinical Science, Intervention and Technology, Karolinska Institutet, SE-14186 Stockholm, Sweden; 6 Department of Physiology, Anatomy and Microbiology, La Trobe University, Bundoora Victoria, 3086 Australia; 7 Research Programs Unit, Molecular Neurology, University of Helsinki, and Folkhälsan Institute of Genetics, 00014 Helsinki, Finland; 8 School of Basic and Medical Biosciences, King’s College London, Guy’s Hospital, London, WC2R 2LS UK

**Keywords:** Reprogramming, Embryology, Pluripotency

## Abstract

Pleomorphic adenoma gene 1 (PLAG1) is a transcription factor involved in cancer and growth. We discovered a *de novo* DNA motif containing a PLAG1 binding site in the promoters of genes activated during zygotic genome activation (ZGA) in human embryos. This motif was located within an Alu element in a region that was conserved in the murine B1 element. We show that maternally provided *Plag1* is needed for timely mouse preimplantation embryo development. Heterozygous mouse embryos lacking maternal *Plag1* showed disrupted regulation of 1,089 genes, spent significantly longer time in the 2-cell stage, and started expressing *Plag1* ectopically from the paternal allele. The *de novo* PLAG1 motif was enriched in the promoters of the genes whose activation was delayed in the absence of *Plag1*. Further, these mouse genes showed a significant overlap with genes upregulated during human ZGA that also contain the motif. By gene ontology, the mouse and human ZGA genes with *de novo* PLAG1 motifs were involved in ribosome biogenesis and protein synthesis. Collectively, our data suggest that PLAG1 affects embryo development in mice and humans through a conserved DNA motif within Alu/B1 elements located in the promoters of a subset of ZGA genes.

## Introduction

Early preimplantation embryo development is dependent on zygotic genome activation (ZGA)^1,2^. Transcription from the newly formed zygotic genome starts gradually already in the one-cell embryo, and a major increase in transcriptional output, known as major ZGA, takes place during the 2-cell (2c) stage in mice and during the 4c-to-8c transition in humans^1,2^. Both minor and major ZGA are essential for cleavage stage development in the mouse^3,4^ and thus needed for the formation of a blastocyst capable of implanting to the uterine endometrium. Therefore, knowledge about the gene expression program during the first stages of embryonic development and regulation of ZGA will help discover factors controlling pluripotency, lineage differentiation and fertility. This has prompted many studies to map transcriptional programs during preimplantation development^5–10^.

In order to understand regulation of gene expression, precise knowledge of transcription start sites (TSSs) is needed. We have used an RNA-seq technology based on the detection of the 5′ ends of transcripts to map active TSSs during the first three days of human preimplantation development^11^. *De novo* motif calling using the regions around the detected TSSs led to the identification of multiple significant motifs harboring known transcription factor binding sites^7,11^. Here, we studied a *de novo* motif discovered in these analyses containing a putative binding site for the pleomorphic adenoma gene 1 (PLAG1). *PLAG1* encodes a C2H2 zinc finger transcription factor and an oncogene that was first characterized in pleomorphic adenomas of the salivary gland^12^. It belongs to the same protein family as the functionally redundant proto-oncogene PLAG-like 2 (*PLAGL2*) and the imprinted tumor suppressor PLAG-like 1 (*PLAGL1*). Ectopic expression of *PLAG1* and *PLAGL2* resulting from chromosomal translocation events can be found in malignant tumors^13^. Associations with cancer have been the focus of most studies on *PLAG* family transcription factors and consequently, less is known about their role in normal physiology. There are no reports on *PLAG* family genes in preimplantation embryo development or pluripotency. By using our human embryo transcriptome data set^11^, *Plag1* knockout (KO) mice^14^, breeding experiments, single-embryo RNA-seq, and time-lapse analysis of cleavage stage embryo development we show that PLAG1 controls a subset of ZGA genes and is needed for normal cleavage stage embryo development.

## Results

### A *de novo* motif containing a PLAG1 binding site is found in the promoters of human ZGA genes

**Figure 1:**
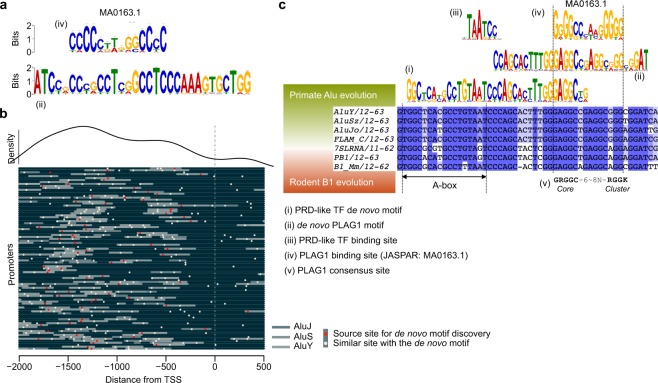
A *De Novo* DNA Motif Containing a PLAG1 Binding Site Is Found in the Promoters of Human ZGA Genes. **(a)** Comparison of (iv) the database PLAG1 binding site (JASPAR MA0163.1) and (ii) the *de novo* PLAG1 motif. **(b)** Location of source sites for the *de novo* PLAG1 motifs (red dots) and similar sites (yellow dots) in the promoters of human ZGA genes. Promoters of the 93 genes containing the motifs are stacked and aligned ranging from 2,000 upstream to 500 downstream bases around transcription start site (TSS, dashed line). The genes are listed in File S1. Locations of AluJ, AluS and AluY elements are highlighted in grey. The curve on top of the figure illustrates the density of the *de novo* PLAG1 motif along the promoter. **(c)** Comparisons of (i) the PRD-like transcription factor *de novo* motif, (ii) the *de novo* PLAG1 motif, (iii) the known PDR-like transcription factor binding site, (iv) the known PLAG1 binding site (JASPAR), and (v) the reported consensus PLAG1 binding site^17^ with human and mouse short interspersed nuclear elements (SINEs). The consensus motif (v) is described by IUPAC notation in which R is a G or A, and K is G or T. The internal RNA polymerase III promoter, A-box, is indicated. The *de novo* motifs and binding motifs are reverse-complemented. Sequences of AluY, AluSz, AluJo, FLAM_C, 7SLRNA, PB1 and B1_Mm were extracted from the Dfam database of repetitive DNA elements. ZGA, zygotic genome activation; TF, transcription factor.

Analysis of the TSSs upregulated during major ZGA in human embryos revealed a significant 31-bp *de novo* DNA motifs harboring a known PLAG1 binding site (Fig. 1a)^11^. This *de novo* PLAG1 motif was present in 74 of the 129 promoters upregulated during ZGA and highly similar sites were found in additional 19 promoters (Fig. 1b and File S1). The *de novo* PLAG1 motif was located within a conserved region of an Alu element in close vicinity to an RNA polymerase III promoter A-box, and it also partially overlapped with the PRD-like transcription factor motif that we identified in our previous study (Fig. 1c; File S1)^11^. Alu elements are primate-specific retrotransposable short interspersed nuclear elements (SINEs) that evolved from a duplication of the 7SL RNA gene^15^ and their evolutionary counterparts in rodents are the B1 elements^16^. Interestingly, the *de novo* PLAG1 motif resided within a segment of the Alu element that was well conserved in the rodent B1 element (Fig. 1c). The PLAG1 binding site within the Alu and B1 elements was nearly identical to the PLAG1 consensus site^17^ (Fig. 1c). Human and mouse PLAG1 have identical DNA binding domains (Fig. S1). These observations suggest that PLAG1 could be involved in human and mouse ZGA through binding to conserved motifs within Alu and B1 elements.

### *Plag1* deficiency affects reproductive success but not ovarian or uterine function

**Figure 2:**
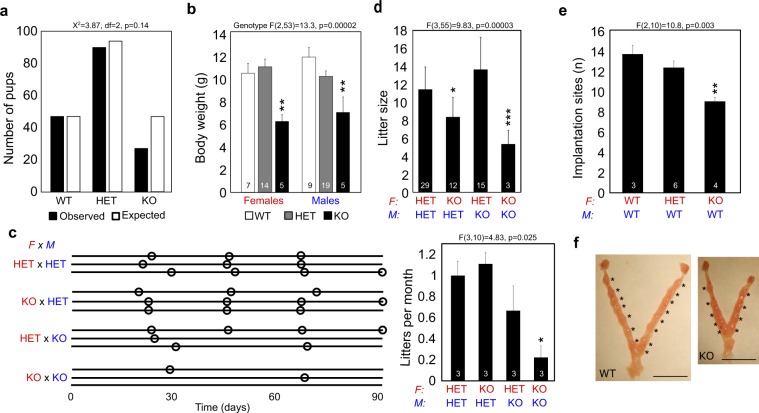
PLAG1 Deficiency Affects Growth and Reproduction in Mice. **(a)** Observed and expected numbers of pup genotypes in litters from *Plag1* heterozygote intercrosses. **(b)** Body weights of female and male pups at weaning (3-weeks-old). **(c)** Frequency of litters from breeding pairs of different genotypes maintained in continuous breeding for 90 days. Every breeding pair is represented with a horizontal line and the birth of a litter is illustrated with a circle. Quantification of the data is shown on the right. **(d)** Mean number of pups in litters from different parent genotypes. **(e)** Mean number of implanted embryos on 7.5–8.5 dpc. **(f)** Representative photos of uteri at dissection. Asterisks indicate implanted embryos. Scale bars are 1 cm. The data in b–e are presented as means + SEM and the number of observations is shown inside the columns. Statistical analysis by χ^2^ test (a) or one-way ANOVA followed by Fisher LSD post-hoc test (**b−f**). ^*^p < 0.05, ^**^p < 0.01, ^***^p < 0.001. F, female; HET, heterozygote; KO, knockout; M, male; WT, wildtype.

To study the role of PLAG1 in fertility and ZGA, *Plag1* KO mice were obtained. The original phenotype was described on the Swiss Webster background whereas our colony was on the CD-1 strain, so we started by studying the reproductive phenotype. Pups from heterozygous intercrosses (HET × HET) did not significantly deviate from the expected Mendelian distribution, although 43% fewer KO pups were born than expected (Fig. 2a). KO pups were 41% smaller than wildtype (WT) at weaning, as reported before (Fig. 2b)^14^. Litter frequency over a three-month continuous breeding period did not differ between HET × HET crosses and pairs consisting of a KO female and a HET male (Fig. 2c). However, KO males with HET females did not manage to maintain the approximate one litter-per-month rate (Fig. 2c). KO × KO intercrosses had significantly reduced litter frequency: the three KO × KO breeding pairs produced only two litters in total during the entire three-month test period (Fig. 2c).

Litter size was affected in couples with KO mothers (Fig. 2d)^14^. Breeding pairs with a KO female and a HET male produced significantly fewer pups per litter, whereas litter size was normal if the parental genotypes were reversed (HET female × KO male) (Fig. 2d). Homozygous KO × KO crosses produced the smallest litters (Fig. 2d). The significant reduction in litter size was seen as early as 7.5–8.5 days *post coitum* (dpc), when fewer implantation marks were observed in KO uteri as compared to HET and WT mothers (Fig. 2e,f). This did not depend on ovarian function, as neither oocyte yield in response to gonadotropin-induced superovulation or ovarian follicle counts differed between WT and KO females (Fig. S2a–d). All normal uterine structures were present in KO females, and body-weight adjusted uterine weights did not differ between the genotypes (Fig. S2e,f). RNA-seq performed on uterine horns, endometrial mucosa and myometrial samples did not reveal differences in transcriptomes between genotypes (Fig. S2g). Taken together, these data show that *Plag1* KO females have no significant defects in their ovaries and uteri and reproduce with normal frequency, but produce significantly fewer pups per litter as compared to HET and WT females.

### Maternal *Plag1* knockout leads to delayed 2-cell stage embryo development, disrupted gene expression, and ectopic expression of paternal *Plag1*

We next studied embryos. Since *Plag1* KO females had small litters regardless of the paternal genotype, we focused on the maternal *Plag1* effect and studied embryos derived from *Plag1* KO females crossed with WT males. These breeding pairs produced HET embryos that lack the maternal *Plag1* allele, and will hereafter be referred to as *matPlag1KO* embryos.

**Figure 3:**
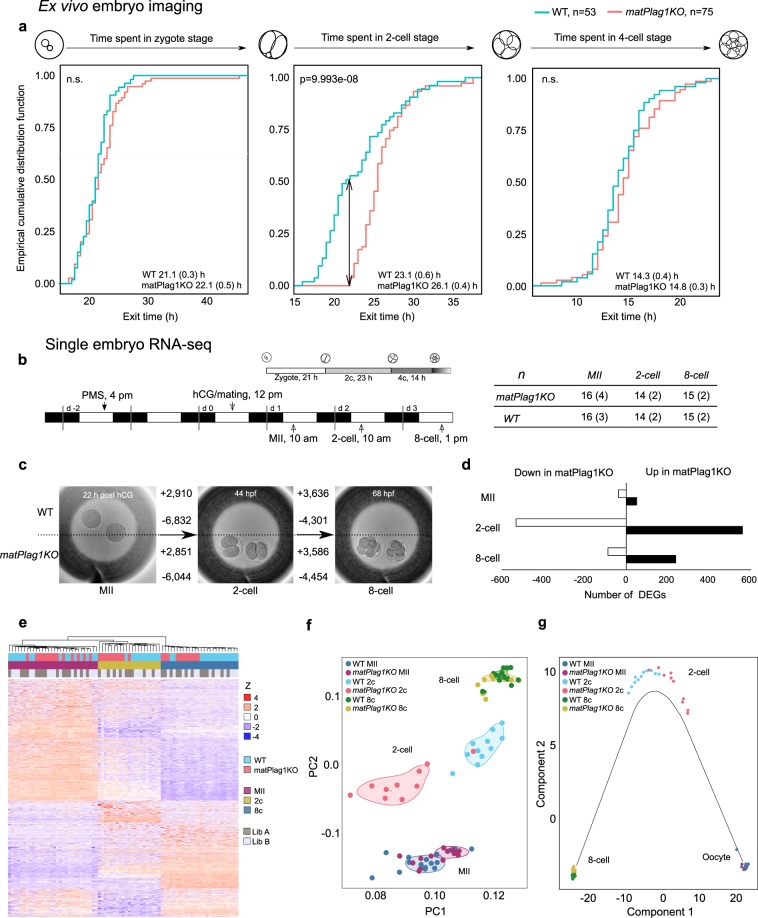
Maternal *Plag1* Knockout Leads to Delayed 2-cell stage Embryo Development and Disrupted Embryonic Gene Expression. **(a)** WT and *matPlag1KO* zygotes were collected for *ex vivo* imaging through time-lapse microscopy and cleavage stage developmental timing was recorded manually for each embryo. Plots show the empirical cumulative distribution function (y-axis, 0–1) of embryos that have exited the corresponding developmental stage at each time point (x-axis, hours). Significance between WT (N = 53) and *matPlag1KO* (N = 75) embryo developmental timing was tested using the Kolmogorov-Smirnov test and p-values are shown in the plots. Average time spent in the stage (SEM) is also shown. **(b)** WT and *matPlag1KO* MII oocytes, 2-cell stage and 8-cell stage embryos were collected for single-embryo RNA-seq. The timing of hormonal treatments, WT embryo development and embryo collection time points are shown together with the numbers of embryos sequenced (numbers of donor females in brackets). **(c)** Number of differentially expressed genes from one developmental stage to another (horizontal arrows) in *matPlag1KO* and WT embryos. **(d)** Number of differentially expressed genes between *matPlag1KO* and WT embryos at the MII oocyte, 2c and 8c stage. **(e–g)** Heatmap (**e**), principal component analysis (**f**), and pseudotime (cell trajectory) analysis (**g**) based on all differentially expressed genes in the libraries. 2c, 2-cell stage; 8c, 8-cell stage; DEG, differentially expressed gene; hCG, human chorionic gonadotropin; KO, knockout; PC, principal component; WT, wildtype.

Preimplantation development of 53 WT and 75 *matPlag1KO* embryos was analyzed by time-lapse microscopy from zygote to late morula during three independent imaging sessions, each of which contained both genotypes (Videos S1 and S2). We discovered that *matPlag1KO* embryos spent significantly more time in the 2c stage compared to WT embryos (Fig. 3a). When 50% of WT embryos had already proceeded to the 4c stage, 100% of *matPlag1KO* embryos were still arrested at the 2c stage (Fig. 3a, arrow). On average, *matPlag1KO* embryos spent 3 h longer in 2c stage compared to WT. The survival of the embryos did not differ between genotypes: 60% of the WT zygotes and 73% of *matPlag1KO* zygotes developed to a morula.

Next, altogether 45 WT and 45 *matPlag1KO* oocytes and embryos representing three developmental stages (oocytes, 2c stage and 8c stage) were collected for single-embryo RNA-seq (Fig. 3b). Spike-in RNA was used for data normalization to correct for the large general changes in cellular RNA content during preimplantation development (Fig. S3). The majority of differentially expressed genes (DEGs) were downregulated in oocyte-to-2c stage transition in both WT (6,823 DEGs down *vs* 2,910 up) and *matPlag1KO* (6,044 DEGs down *vs* 2,851 up) embryos reflecting degradation of maternal transcripts (Fig. 3c). More DEGs were upregulated in the 2c–8c transition, when the zygotic genome becomes fully active (3,636 DEGs up in WT and 3,586 in *matPlag1KO*) (Fig. 3c).

The largest difference between WT and *matPlag1KO* embryos was found at the 2c stage when *matPlag1KO* embryos had 530 downregulated and 559 upregulated DEGs compared to WT, suggesting a dysregulation of approximately 11% of all genes regulated during the oocyte-to-2c transition (Fig. 3c,d). The differences were also clearly detected in different clustering analyses. Heatmap clustered the embryos into three primary groups by developmental stage (oocyte, 2c, 8c) but also by genotype at the 2c stage (Fig. 3e). Principal component analysis likewise separated the embryos primarily by developmental stage and by genotype at the 2c stage (Fig. 3f). Cell trajectory analysis yielded the same result and further suggested that the transcriptional program in the *matPlag1KO* embryos lagged behind the WT at the 2c stage (Fig. 3g).

**Figure 4:**
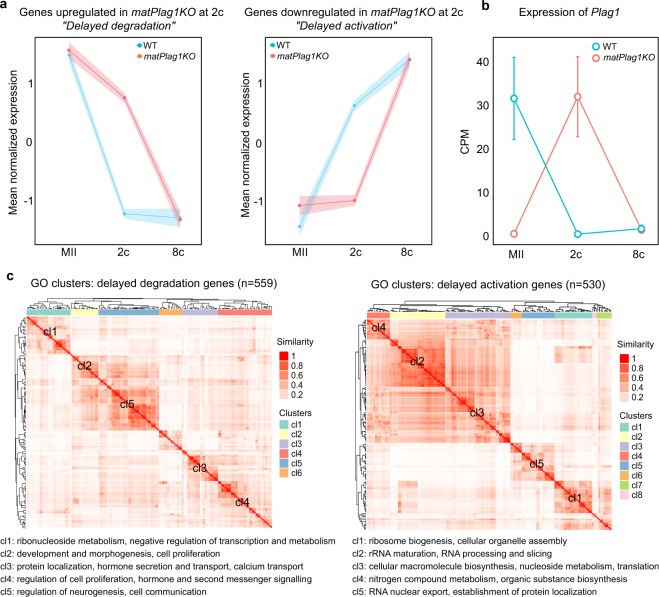
*MatPlag1KO* 2-Cell Embryos Have Delayed Regulation of Two Functionally Differing Sets of Genes and They Ectopically Express *Plag1* from the Paternal Allele. **(a)** Mean normalized expression (Z) pattern of the 559 genes that are significantly upregulated (“delayed-degradation”) and the 503 genes that are significantly downregulated (“delayed-activation”) in *matPlag1KO* embryos compared to WT embryos at the 2c stage. **(b)** Normalized expression of *Plag1* in the MII oocyte, 2c and 8c stages. **(c)** Heatmaps displaying semantic similarity among the top-150 significantly enriched GO terms associated to the delayed-degradation (left) and delayed-activation (right) genes. Five largest clusters are depicted (cl1–cl5), and common denominators among GO terms belonging to these clusters are shown. The full GO lists are provided in File S2. 2c, 2-cell stage; 8c, 8-cell stage; CPM, counts per million; GO, gene ontology; KO knockout; WT, wildtype.

The expression pattern of the dysregulated DEGs in 2c *matPlag1KO* embryos was studied by plotting their mean expression levels from oocyte to the 8c stage. DEGs upregulated in *matPlag1KO* embryos compared to WT were actually maternally loaded transcripts whose degradation was delayed (Fig. 4a). Similarly, the DEGs downregulated in *matPlag1KO* embryos at the 2c stage were in fact genes whose upregulation was delayed (Fig. 4a). We hereafter refer to these two sets of genes as “delayed-degradation” and “delayed-activation” genes. By the 8c stage, their expression levels had reached the WT levels (Fig. 4a). *Plag1* transcripts were present in WT oocytes but absent in KO oocytes (Fig. 4b). In WT embryos, the maternally provided *Plag1* was completely degraded by the 2c stage and remained absent in 8c embryos, suggesting that *Plag1* is normally not expressed from the zygotic genome (Fig. 4b). Interestingly, in clear contrast to WT embryos, *matPlag1KO* embryos expressed *Plag1* in the 2c stage. As these embryos lack maternal *Plag1* allele, the expression must stem from activation of the paternal allele. This ectopic expression of *Plag1* in the 2c stage *matPlag1KO* embryos returned to baseline by 8c stage (Fig. 4b), when also the delayed-activation and delayed-degradation genes had caught up with the WT expression levels (Fig. 4a). This may suggest rescue of the ZGA by paternal *Plag1* expression. Factors that are needed for ZGA are maternally provided into the oocyte during folliculogenesis. Our data shows that *Plag1* transcripts were maternally provided into mouse oocytes (Fig. 4b) and they were already present during folliculogenesis (Fig. S5). In addition, analysis of two independent human RNA-seq datasets showed that *PLAG1* is maternally provided to human oocytes as well with decreasing levels towards blastocyst stage (Fig. S4).

Gene ontology analyses showed that the delayed-activation genes belong into categories relevant to ribosome biogenesis, RNA processing, and translation, whereas the delayed-degradation genes clustered to diverse GO categories ranging from neurogenesis to negative regulation of transcription (Fig. 4c, File S2). The delayed-activation GOs showed higher similarity to genes typically upregulated during 2c–8c transition in WT embryos (best-match average (BMA) 0.792) than to those downregulated (BMA 0.531) (File S3). Conversely, the delayed-degradation GOs showed higher similarity to genes normally downregulated between 2c–8c stages (BMA 0.714) than to those upregulated (BMA 0.445) (File S3). These data confirmed that embryos that lacked the maternal *Plag1* were characterized by delayed transition through the 2c stage due to specific sets of genes being dysregulated compared to WT embryos.

### The delayed-activation genes overlap with human ZGA genes and have enriched PLAG1 *de novo* motif in their promoters

**Figure 5:**
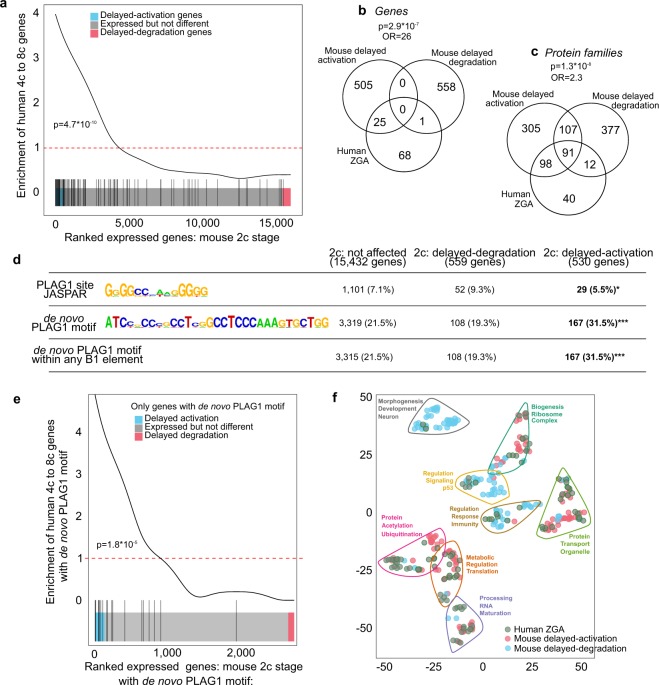
*MatPlag1KO* Delayed-Activation Genes Overlap with Human ZGA Genes, Have Enriched PLAG1 *De Novo* Motif in Their Promoters, and Associate with Protein Synthesis. **(a)** Gene set enrichment analysis comparing genes that are expressed in mouse embryos (WT and *matPlag1KO*) at the 2c stage (x-axis) with the genes upregulated during human ZGA (4c–8c transition) (y-axis). The black vertical lines shows the location of orthologous human genes among the ranked mouse genes, and the curve depicts the enrichment. Red dotted line indicates “no enrichment” level. Significance was tested with the GeneSet test function. **(b**,**c)** Venn diagram showing the overlap between delayed-activation and delayed-degradation genes (**b**) and proteins (**c**) with the human ZGA genes and proteins. **(d)** Presence of database PLAG1 binding sites (JASPAR), *de novo* PLAG1 motifs, and *de novo* PLAG1 motifs within B1 elements in the promoters of genes expressed in mouse 2c embryos. Total number of genes in different categories as well as number of unique genes with the motif within −2,000 to +500 of their TSS are shown. Enrichment over not affected genes was analyzed with Fisher’s exact test. ^*^p < 0.05, ^***^p < 0.001. (**e**) Gene set enrichment analysis comparing human and mouse genes that contain at least one *de novo* PLAG1 motif in their promoters. Significance was tested with the GeneSet test function. (**f**) t-SNE plot demonstrating similarity among the top-100 GO categories associated to human ZGA, mouse delayed-activation and mouse delayed-degradation genes that contain at least one *de novo* PLAG1 motif in their promoters (within −2,000 to +500 bp of TSS). The eight largest clusters with the most common words within the clusters are shown. 2c, 2-cell stage; 4c, 4-cell stage; 8c, 8-cell stage; GO, gene ontology; KO, knockout; OR, odds ratio; TSS, transcription start site; WT, wildtype.

We next wanted to compare our current mouse embryo data with our earlier human embryo transcriptome data^11^, and converted the mouse genes to human orthologues. The gene expression changes during major ZGA in humans (4c–8c transition) and WT mice (2c–8c transition) were highly similar in general between our datasets (Fig. S6). A gene set analysis showed that the genes upregulated during human major ZGA were significantly enriched among the mouse delayed-activation genes (Fig. 5a). The same result was obtained using the χ^2^ test; a significant overlap was found between the human major ZGA genes with the *matPlag1KO* delayed-activation genes but not with the delayed-degradation genes (Fig. 5b). Comparison of protein families yielded similar results (Fig. 5c).

We then studied the occurrence of the database PLAG1 binding sites and the *de novo* PLAG1 motifs in the affected mouse gene promoters. Database PLAG1 binding sites were found in less than 10% of the promoters in general, and surprisingly the occurrence was significantly lower in the promoters of the delayed-activation genes (5.5%) (Fig. 5d). However, the results were the opposite when we used our *de novo* PLAG1 motif instead. A significantly higher proportion of the delayed-activation promoters contained at least one *de novo* PLAG1 motif (31.5%) compared to “expressed but not affected” (21.5%) and delayed-degradation (19.3%) genes (Fig. 5d, File S4). We also studied the connection of these *de novo* PLAG1 motifs to B1 elements and obtained virtually identical results, showing that the vast majority of the *de novo* PLAG1 motifs reside within B1 elements (Fig. 5d). In addition to occurrence (present or not), we also considered the frequency (how many) of the *de novo* PLAG1 motifs. A significantly higher frequency was found in the promoters of delayed-activation genes compared to delayed-degradation genes (Fig. S7).

Finally, we compared the homologous mouse delayed-activation and human ZGA genes that contained at least one *de novo* PLAG1 motif and found a significant overlap (Fig. 5e). We GO-annotated the three gene groups, *i*.*e*. human ZGA, mouse delayed-activation and mouse-delayed degradation genes. Hierarchical clustering revealed that human ZGA and mouse delayed-activation genes clustered together to categories representing ribosomes, protein transport, translation, RNA processing, and protein metabolism, whereas mouse delayed-degradation genes showed little overlap with the human ZGA clusters (Fig. 5f). These data suggest that mouse and human embryos have a functionally conserved set of genes that is activated during ZGA and contain PLAG1 binding sites within repetitive elements in their promoters.

## Discussion

In the present study, we have discovered a new role for the oncogene *PLAG1* in the regulation of ZGA. We identified a *de novo* assembled motif containing a PLAG1 binding site among the promoters upregulated during ZGA in human embryos, and showed that the lack of maternally loaded *Plag1* in mouse oocytes lead to a significant delay in ZGA on a transcriptional level with consequences for the timing of cleavage-stage development. Restoration of gene expression levels and embryo developmental speed coincided with ectopic expression of *Plag1* from the paternal allele. These data imply a functional role for PLAG1 in the regulation of ZGA. Our data further propose that PLAG1 target genes have roles in central cellular processes that relate to ribosomes, RNA and protein metabolism, which undoubtedly have an essential function during early embryo growth.

We show that *matPlag1KO* embryos that lack the *Plag1* transcript only during the first hours following fertilization spent significantly longer time in the 2c stage compared to WT embryos. The 2c stage is the developmental stage when the major ZGA takes place in the mouse, and failure to activate transcription leads to developmental arrest^3^. Embryo development can be affected by single genes. For example, knockdown of the pluripotency factor *Lin28* in mouse embryos leads to arrest at the 2c–4c stage^18^, and KO of the maternal-effect gene *Mater* to arrested embryonic development at the 2c stage^19^. At the 2c stage, when the development of every studied *matPlag1KO* embryo was delayed compared to WT, these embryos started expressing *Plag1* from the paternal allele. This upregulation was followed by regaining the normal developmental pace as well as normal transcriptomic program by the 8c stage, suggesting that the ectopic paternal expression of *Plag1* rescued the embryo phenotype. Although these data argue that *Plag1* is essential for embryo development at the 2c stage, we noted that even homozygous *Plag1* KO intercrosses occasionally produced litters, showing that a complete lack of *Plag1* is not strictly lethal to the embryos. Analysis of embryos from KO × KO breedings would help to understand the preimplantation phenotype. Unfortunately, the extremely poor breeding success of the KO × KO couples renders these studies unpractical. We further hypothesize that *Plagl2*, the *Plag1 -*family member with redundant functions to *Plag1* that is also maternally provided, might rescue embryo development in some cases. Testing this hypothesis would require generation of *Plag1/Plagl2* double KO mice, which is impossible due to the severe phenotype of *Plagl2* KO mice; KO pups die shortly after birth to starvation due to their inability to absorb chylomicrons^20^.

Although *matPlag1KO* embryos regained a normal developmental pace after exiting the 2c stage, their developmental success was not the same as that of WT embryos, as evidenced by the reduced number of implantation sites and smaller litter size. Our embryo time-lapse data do show that *matPlag1KO* embryos developed to the blastocyst stage with equal efficiency as WT embryos. Since KO uteri already showed less implantation marks at 7.5−8.5 dpc, the embryo loss must occur sometime between 4.5 and 7.5 dpc, *i*.*e*., when the blastocyst normally implants or shortly thereafter. Developmental competence of human embryos can be scored after *in vitro* fertilization through morphokinetic measurements, and the time an embryo spends in the 2c and 4c stages is a significant determinant of developmental potential^21^. Following this, we hypothesize that the delayed 2c stage development and associated dysregulation of over 1,000 genes in *matPlag1KO* embryos could have adverse effects on the overall developmental potential of the blastocysts. In addition, synchronous preparation of both the embryo and endometrium for implantation is a prerequisite for successful implantation during the window of receptivity. Therefore, a simple delay in preimplantation development could contribute to some blastocysts missing this critical window^22^.

The *de novo* PLAG1 motif frequently localized within repetitive elements in the promoters of the ZGA genes. Alu elements are transposable elements ubiquitously present in primate genomes with involvement in gene regulation through various mechanisms^23^ and their counterparts in the mouse genome are B1 elements^16^. Despite independent evolution for over 80 million years, the densities of Alu and B1 elements in promoters of orthologous genes in humans and mice are surprisingly correlated^15,24,25^. In particular, Alu and B1 elements are highly enriched in promoters that are activated during ZGA^26^. The fact that the region that contains the PLAG1 binding site is conserved within the Alu and B1 elements suggests positive evolutionary selection. Collectively, these findings may suggest that Alu and B1 play a role in ZGA in humans and mice by attracting transcription factors such as PLAG1 to gene promoters.

The delayed-activation genes were mainly related to ribosome biogenesis, maturation and function by ontology. Even when we restricted the analysis to those genes that contained the *de novo* PLAG1 motif, GO analyses suggested roles in ribosome biogenesis and RNA and protein metabolism, both in mice and humans. It has been shown that Alu elements are enriched in promoters of genes involved in ribosome biogenesis, protein biosynthesis and RNA metabolism^25^, and one function could be to attract transcription factors such as PLAG1. Many oncogenes have effects on ribosome biogenesis, which enables cancerous cells to increase protein synthesis and grow rapidly^27^. Although *PLAG1* is an oncogene, its potential role in ribosome biogenesis and protein synthesis has not been investigated. One of the most striking phenotypes of the *Plag1* KO mice is their small size^14^. In addition, *PLAG1* polymorphisms associate with body size and growth in farm animals and humans^13,28–31^. Based on our data, we present a hypothesis that PLAG1*-*associated growth phenotypes, such as growth retardation in the KO mice, results from modulation of protein synthesis that affects cell size and division rate.

We conclude that the lack of maternally provided *Plag1* leads to a delay in ZGA manifested as prolonged 2c stage development, which is rescued by ectopic paternal *Plag1* expression. This delay and associated dysregulation of genes needed for ribosome biogenesis, RNA and protein metabolism could lead to reduced embryo competence for implantation, explaining the reduced litter size in KO mothers. We further propose that the effect of PLAG1 on ZGA genes arose through retrotransposition of Alu and B1 elements, where the PLAG1 binding sites have been under positive selection. Follow-up studies should focus on a deeper analysis of PLAG1 involvement in protein synthesis, as this is a mechanism that would explain many of the reported biological activities of PLAG1, including tumorigenicity, cell proliferation, and growth.

## Materials and Methods

Detailed materials and methods are provided in the Supplemental Information.

### Mouse studies

All experiments were approved by the Swedish Board of Agriculture (#S5–14) and performed in accordance with the ethical licence.

*Plag1KO* mice^14^ in CD-1 strain were a kind gift from Prof. Wim Van de Ven (University of Leuven, Belgium) and Dr. Carol Schuurmans (University of Calgary, Canada). The colony was established via embryo transfers, maintained as HET breedings, and genotyped using ear punches. To generate oocytes and zygotes for experiments, sexually mature young females were superovulated. Their ovaries and uteri were collected for histology and gene expression analysis. For time-lapse microscopy, zygotes (n = 103 *matPlag1KO* and n = 89 WT) were placed into a live-cell imaging incubator under a Nikon Ti-E spinning disk wide-field microscope and imaged every 30 min with an Andor EM-CCD camera. The experiment was carried out three times with both genotypes present, and the developmental pace was manually scored from the images by a researcher blinded to the genotypes.

To count implantation marks, KO and WT females were mated with trained WT studs and their uteri were collected 7.5–8.5 dpc for visual inspection. The experiment was carried out twice, with a total of 8 KO and 8 WT females.

RNA expression in uterus samples (n = 8 *Plag1KO*, n = 8 WT) was measured using STRT RNA-seq protocol^32^. Uterine horns were cut longitudinally, mucosa and myometrium separated by scraping, and the samples stored in RNAlater (Ambion, Foster City, CA, USA) until RNA extraction (RNeasy Mini kit, Qiagen, Hilden, Germany). Ten nanograms of RNA (RIN > 8) was used for RNA-seq as described^32^.

RNA expression in single manually picked oocytes and embryos was detected by analyzing two independent libraries prepared on three occasions with both genotypes present both times. Modified STRT protocol^32^ was used.

### RNA-seq data analysis

RNA-seq data was analyzed as described previously^32^. Libraries with a median gene expression of log2 counts per million (cpm) under 0 were excluded, and cell libraries were normalized with the TMM methods using EdgeR^33^. Differential gene expression analysis using EdgeR was performed on genes that had 1 cpm in at least five or more samples. Principal component analysis, heatmaps, hierarchical clustering, cell trajectory and GO analyses were carried out using DEGs in R^34^. Enriched GO terms were identified using Fisher statistics and compared via semantic similarity analysis using the Wang algorithm^35^. Homologene from NCBI was used to convert the human genes to the homologous mouse genes, and gene enrichment analyses were carried out using geneSetTest function from the *limma* package^36^.

### Promoter analyses

Human embryo promoter analysis was performed as previously described^11^. SINE repetitive elements were retrieved from the Dfam database^37^ and aligned in JalView2. Mouse embryo promoter analysis was carried out with Homer^38^. Distance of repetitive elements and *de novo* PLAG1 motifs to the nearest TSS was plotted using ggplot2. Enrichment was analyzed using Fisher’s exact test.

### Statistical analysis

Continuous data were analyzed with Student’s *t*-test, one-way or two-way ANOVA, followed by Fisher LSD post-hoc test. Normality was tested with the Shaphiro-Wilks test and homoscedasticity with Levene’s test. Categorical data were analyzed with the χ^2^ test. The exit time of embryos from each developmental stage was plotted as an empirical distribution function (ecdf) using ggplot2 and significance tested using the Kolmogorov-Smirnov test. All analyses were carried out using R, the p values are two-tailed and considered significant if p < 0.05.

## Supplementary information

Video S1

Video S2

Supplementary information

File S1

File S2

File S3

File S4

## Data Availability

RNA-seq data have been deposited to Gene Expression Omnibus data repository as a SuperSeries record under the reference GSE111040.

## References

[1] Jukam D, Shariati SAM, Skotheim JM (2017). Zygotic Genome Activation in Vertebrates. Dev Cell.

[2] Niakan KK, Han J, Pedersen RA, Simon C, Pera RA (2012). Human pre-implantation embryo development. Development.

[3] Warner CM, Versteegh LR (1974). *In vivo* and *in vitro* effect of alpha-amanitin on preimplantation mouse embryo RNA polymerase. Nature.

[4] Abe KI (2018). Minor zygotic gene activation is essential for mouse preimplantation development. Proc Natl Acad Sci USA.

[5] De Iaco A (2017). DUX-family transcription factors regulate zygotic genome activation in placental mammals. Nat Genet.

[6] Petropoulos S (2016). Single-Cell RNA-Seq Reveals Lineage and X Chromosome Dynamics in Human Preimplantation Embryos. Cell.

[7] Töhönen, V. *et al*. Transcription Activation Of Early Human Development Suggests DUX4 As An Embryonic Regulator. *bioRxiv*, 10.1101/123208 (2017).

[8] Vassena R (2011). Waves of early transcriptional activation and pluripotency program initiation during human preimplantation development. Development.

[9] Xue Z (2013). Genetic programs in human and mouse early embryos revealed by single-cell RNA sequencing. Nature.

[10] Yan L (2013). Single-cell RNA-Seq profiling of human preimplantation embryos and embryonic stem cells. Nat Struct Mol Biol.

[11] Tohonen V (2015). Novel PRD-like homeodomain transcription factors and retrotransposon elements in early human development. Nat Commun.

[12] Kas K (1997). Promoter swapping between the genes for a novel zinc finger protein and beta-catenin in pleiomorphic adenomas with t(3;8)(p21;q12) translocations. Nat Genet.

[13] Juma AR, Damdimopoulou PE, Grommen SV, Van de Ven WJ, De Groef B (2016). Emerging role of PLAG1 as a regulator of growth and reproduction. J Endocrinol.

[14] Hensen K (2004). Targeted disruption of the murine Plag1 proto-oncogene causes growth retardation and reduced fertility. Dev Growth Differ.

[15] Ullu E, Tschudi C (1984). Alu sequences are processed 7SL RNA genes. Nature.

[16] Labuda D, Sinnett D, Richer C, Deragon JM, Striker G (1991). Evolution of mouse B1 repeats: 7SL RNA folding pattern conserved. J Mol Evol.

[17] Voz ML, Agten NS, Van de Ven WJ, Kas K (2000). PLAG1, the main translocation target in pleomorphic adenoma of the salivary glands, is a positive regulator of IGF-II. Cancer Res.

[18] Vogt EJ, Meglicki M, Hartung KI, Borsuk E, Behr R (2012). Importance of the pluripotency factor LIN28 in the mammalian nucleolus during early embryonic development. Development.

[19] Tong ZB (2000). Mater, a maternal effect gene required for early embryonic development in mice. Nat Genet.

[20] Van Dyck F (2007). Loss of the PlagL2 transcription factor affects lacteal uptake of chylomicrons. Cell Metab.

[21] Wong CC (2010). Non-invasive imaging of human embryos before embryonic genome activation predicts development to the blastocyst stage. Nat Biotechnol.

[22] Wang H, Dey SK (2006). Roadmap to embryo implantation: clues from mouse models. Nat Rev Genet.

[23] Elbarbary RA, Lucas BA, Maquat LE (2016). Retrotransposons as regulators of gene expression. Science.

[24] Mouse Genome Sequencing C (2002). Initial sequencing and comparative analysis of the mouse genome. Nature.

[25] Polak P, Domany E (2006). Alu elements contain many binding sites for transcription factors and may play a role in regulation of developmental processes. BMC Genomics.

[26] Ge SX (2017). Exploratory bioinformatics investigation reveals importance of “junk” DNA in early embryo development. BMC Genomics.

[27] Pelletier J, Thomas G, Volarevic S (2018). Ribosome biogenesis in cancer: new players and therapeutic avenues. Nat Rev Cancer.

[28] Fink T (2017). Functional confirmation of PLAG1 as the candidate causative gene underlying major pleiotropic effects on body weight and milk characteristics. Sci Rep.

[29] Rubin CJ (2012). Strong signatures of selection in the domestic pig genome. Proc Natl Acad Sci USA.

[30] Utsunomiya YT (2013). Genome-wide association study for birth weight in Nellore cattle points to previously described orthologous genes affecting human and bovine height. BMC Genet.

[31] Zhang W (2016). Multi-strategy genome-wide association studies identify the DCAF16-NCAPG region as a susceptibility locus for average daily gain in cattle. Sci Rep.

[32] Krjutskov K (2016). Single-cell transcriptome analysis of endometrial tissue. Hum Reprod.

[33] Robinson MD, McCarthy DJ, Smyth GK (2010). edgeR: a Bioconductor package for differential expression analysis of digital gene expression data. Bioinformatics.

[34] R: A language and environment for statistical computing (R Foundation for Statistical Computing, Vienna, Austria 2010).

[35] Yu G (2010). GOSemSim: an R package for measuring semantic similarity among GO terms and gene products. Bioinformatics.

[36] Ritchie ME (2015). limma powers differential expression analyses for RNA-sequencing and microarray studies. Nucleic Acids Res.

[37] Hubley R (2016). The Dfam database of repetitive DNA families. Nucleic Acids Res.

[38] Heinz S (2010). Simple combinations of lineage-determining transcription factors prime cis-regulatory elements required for macrophage and B cell identities. Mol Cell.

