# Supplementary material for: Pleomorphic Adenoma Gene 1 Is Needed For Timely Zygotic Genome Activation and Early Embryo Development
Source: Sci Rep. 2019 Jun 10;9:8411. doi: 10.1038/s41598-019-44882-0 (PMC6557853; doi:10.1038/s41598-019-44882-0)
Supplement: Supplementary file 3 — Supplementary information [file 41598_2019_44882_MOESM3_ESM.pdf]

Supplemental information

Pleomorphic Adenoma Gene 1 Is Needed For Timely Zygotic Genome Activation and Early Embryo Development

Elo Madissoon*¤, Anastasios Damdimopoulos*, Shintaro Katayama*, Kaarel Krjutskov, Elisabet Einarsdottir, Katariina Mamia, Bert De Groef, Outi Hovatta, Juha Kere¤, Pauliina Damdimopoulou¤

*Equal contribution
¤Corresponding authors elo.madissoon@ebi.ac.uk; juha.kere@ki.se; pauliina.damdimopoulou@ki.se

INDEX

Supplemental Materials and Methods **Page 3**
Video S1 (a separate attachment), legend **Page 5**
Time-lapse video of WT embryo development
Video S2 (a separate attachment), legend **Page 5**
Time-lapse video of *matPlag1KO* embryo development
File S1 (a separate attachment), legend **Page 5**
de novo PLAG1 motifs and repetitive elements in human ZGA genes
File S2 (a separate attachment), legend **Page 5**
GO clusters associated with delayed-activation and delayed-degradation genes
File S3 (a separate attachment), legend **Page 6**
GO clusters associated with up- and downregulated genes during ZGA in WT mice
File S4 (a separate attachment), legend **Page 6**
de novo PLAG1 motifs in mouse ZGA genes
Figure S1 **Page 7**
Comparison of human PLAG1 and mouse Plag1 protein sequence
Figure S2 **Page 8**
Plag1 deficiency does not have significant effects on ovaries and uterus
Figure S3 **Page 9**
Size of the mRNA and spike-in reference RNA libraries
Figure S4 **Page 10**
Expression of *PLAG1* in human embryos
Figure S5 **Page 11**
Expression of *Plag1* transcripts in mouse ovary as shown by X-gal staining
Figure S6 **Page 12**
Gene set comparison of mouse and human ZGA genes
Figure S7 **Page 13**
Frequency of *de novo* PLAG1 motifs and B1 elements in delayed-activation and delayed-degradation gene promoters
References **Page 14**

Supplemental Materials and Methods

Mouse studies

The animals were housed in standard conditions (19−21°C, 55% humidity, lights 6:00 am−6:00 pm) with free access to feed (irradiated Global 18% diet 2918; Envigo, Huntingdon, UK) and tap water. In all experiments, animals were sacrificed by cervical dislocation.

Genotyping: Ear punches were used for genotyping with primers Plag1MT_F (5'-CAGTTCCCAGGTGTCCAACAAG-3'), Plag1MT_R (5'-AATGTGAGCGAGTAACAACCCG-3'), Plag1WT_F (5'-CGGAAAGACCATCTGAAGAATCAC -3'), and Plag1WT_R (5'-CGTTCGCAGTGCTCACATTG-3').

Superovulation: Sexually mature 1–3-months-old *Plag1* KO and WT females were superovulated by i.p. injection of 5 IU pregnant mare serum (Folligon; Intervet, Dublin, Ireland), followed two days later by 5 IU human chorionic gonadotropin (hCG) (Chorulon; Intervet, Dublin, Ireland), and mated with trained WT studs. MII oocytes (no mating) and zygotes were collected from oviducts the following morning. Cumulus cells were removed with hyaluronidase (0.3 mg/ml, Sigma-Aldrich, St.Louis, MO, USA).

Time-lapse imaging For time-lapse microscopy, zygotes were placed 2−4 per well into Primo Vision embryo culture dishes (Vitrolife, Göteborg, Sweden) under a Nikon Ti-E spinning disk wide-field microscope with a live-cell imaging incubator and imaged every 30 min with an Andor EM-CCD camera for 90 h 30 min. The imaging was repeated three times with embryos from 5 KO females (providing 103 embryos) and 6 WT females (providing 89 embryos) with both genotypes present at every session. Developmental pace was manually scored from the images by a researcher blinded to the genotypes.

Ovary and uterus analyses Ovaries and uteri were collected during superovulation experiments, their weights recorded, and tissues stored in 4% (w/v) paraformaldehyde. Paraffin-embedded tissue was processed to 4-μm hematoxylin-eosin-stained sections and slides digitalized with a Mirax Slide Scanner (Zeiss, Göttingen, Germany). Transverse sections from the middle of the uterine horn were used for histological examination. One section from the middle of the ovary was used for assessment of follicles in different developmental categories (preantral, antral, atretic and corpora lutea) and their number adjusted for the ovary surface area using Pannoramic Viewer software (3DHistech, Budapest, Hungary). Altogether, 22 KO and 13 WT animals were used for assessment of the ovary, and 8 KO and 8 WT for uterus histology. For X-gal staining, ovaries were collected from 3 KO and 3 WT females, aged between 4 and 5 months, fixed in 4% paraformaldehyde, cryosectioned at 12 μm and subjected to X-gal staining as described before [1], with the following modifications: sections were placed in X-gal solution at room temperature immediately without thawing; after overnight incubation in X-gal solution, the tissues were not postfixed; and eosin was used as a counterstain.

Embryo RNA-seq Zygotes collected from superovulated females were treated with hyaluronidase and placed in KSOM medium (EMD Millipore, Nottingham, UK) under ovoil-100 (Vitrolife) in IVF 4-well plates (Sigma-Aldrich) for culture at 37°C under 5% CO2/95% air. Single embryos or uncultured MII oocytes were picked into 4 μl library lysis buffer containing 5 mM Tris-HCl pH 8.0 (Sigma-Aldrich), 2 mM dNTP mixture (ThermoFisher Scientific, Waltham, MA, USA), 10 mM DTT (Sigma-Aldrich), 0.05% Triton X-100 (Sigma-Aldrich), 400 nM anchored oligo(dT) primer biotin–TTAAGCAGTGGTATCAACGCAGAGTCGAC$(T)_{29}$V where V is a LNA nucleotide (Exiqon, Vedbaek, Denmark), and 4 U RiboLock RNase inhibitor (ThermoFisher). Embryos were picked as shown in Figure 3b on three different occasions, yielding a total of 16 zygotes, 14 2c-stage embryos, and 15 8c-stage embryos for both *matPlag1KO* and WT. Two separate 46-plex libraries (libA and libB) were made as

described previously [2] with the following modifications: barcoded 10 μM template-switching oligonucleotides were added prior to reverse transcriptase, ERCC spike-in Mix A was diluted 15,300-fold with clean water, and 1 μl was taken per library reverse transcriptase master mix. Twenty cycles of PCR were used for the first round of amplification and ten additional cycles for the second round to introduce Illumina-compatible universal sequences. Both libraries contained all developmental stages and genotypes.

RNA-seq data analysis

The reads were filtered, samples de-multiplexed, UMIs joined, reads trimmed and mapped to the reference mouse genome mm9 by TopHat [3]. The resulting bam files were converted to tag directories employing Homer [4] and were subsequently used to estimate the reads in all annotated genes. Annotations were in GTF file format retrieved from UCSC and were concatenated to a GTF file with the ERCC annotations. Gene counts were then imported to R [5] and libraries with a median gene expression of log2 counts per million (cpm) under 0 were excluded from further analysis. Cell libraries, excluding the ERCC spike-in counts, were normalized with EdgeR [6] using the TMM normalization method. The ERCC counts were used for normalization between the various embryonic cell stages by scaling the library sizes. EdgeR was also employed for the subsequent differential gene expression analysis, which was performed on genes that had 1 cpm in at least five or more samples and the rest of the genes were filtered out. After removal of low-expressed and unexpressed genes, the gene counts were renormalized. Principal component analysis was performed in R by using the genes that were significant in any of the comparisons between the genotypes. Heatmaps were plotted on TMM-normalized counts exported from EdgeR and gene expression was standardized across all samples (mean = 0 and SD = 1). Samples and genes were clustered using hierarchical clustering in R and plotted employing the ComplexHeatmap library [7]. The same gene set was used for the cell trajectory (pseudotime) analysis by the monocle package [8]. Gene ontology analysis was carried out in R with the topGO library. To identify enriched GO terms, the classic algorithm and Fisher statistic were used and analysis was carried out on up- and downregulated genes separately. Semantic similarity between the GO terms was calculated using the Wang algorithm in the GOSemSim bioconductor package [9]. The result is given as the best-match average (BMA) score that ranges from 0 to 1. Gene set enrichment analysis was conducted to test whether the mouse genes homologous to the human genes regulated between the 4c and 8c stage were also regulated in mouse development. To calculate the p values, the geneSetTest function from the *limma* package [10] was used. The moving average of the enrichment was calculated with the tricubeMovingAverage function and plotted with ggplot2. Homologene from NCBI was used to convert the human genes to the homologous mouse genes. The significance in overlap of the human genes with the mouse genes regulated in the KO at the 2c stage was calculated with the Fisher test in R. Genes were also converted to protein families using the bioconductor libraries for genome-wide annotation for human and mouse (org.Hs.eg.db/org.Mm.eg.db). For genes that had more than one protein family annotated to them, only one of the protein families was used in order not to inflate the number of overlapping or non-overlapping families between the different gene groups.

Promoter analyses

Human embryo promoter analysis was performed as previously described [11]. Briefly, the *de novo* motif was compared with known motifs by TomTom [12]. We applied MEME [13] for motif analysis within the upregulated promoters, and identified sequences similar to the PLAG1 motif (MA0163.1 in JASPAR) [14,15] using MAST [16]. The location of Alu elements within the promoters was based on the RepeatMasker track in the UCSC Genome Browser. Human

and mouse SINE repetitive elements DF0000002 (AluY), DF0000051 (AluSz), DF0000034 (AluJo), DF0000144 (FLAM_C), DF0000016 (7SLRNA), DF0003101 (PB1) and DF0001733 (B1_Mm) were retrieved from the Dfam database [17] and aligned and highlighted according to the percent identity by JalView2. Mouse embryo promoter analysis was carried out with Homer [4]. The hits of the repetitive elements and the motifs were imported into R. Repetitive elements that fell within the promoter regions (TSS -2,000 bp to +500bp) were kept and the distance to the nearest TSS calculated for the repeats and the *de novo* PLAG1 motifs and plotted using ggplot2. Enrichment was analyzed using Fisher's exact test.

Video S1. Time-lapse video of wildtype (WT) embryo development
A representative movie of WT mouse embryo development from zygote to morula. The video time line is indicated (0-89 h) as well as the time spent in 2-cell stage.

Video S2. Time-lapse video of *matPlag1KO* embryo development
A representative movie of *matPlag1KO* mouse embryo development from zygote to morula. The video time line is indicated (0-89 h) as well as the time spent in 2-cell stage.

File S1. *de novo* PLAG1 motifs and repetitive elements in human zygotic genome activation (ZGA) genes

Sheet MEME Position of source sequences for the *de novo* PLAG1 motif in human ZGA transcript far 5' ends (TFEs) within –2,000 bp upstream to 500 bp downstream from the transcription start site (TSS). The sheet gives the MEME [13] analysis results for human ZGA TFEs that were used to derive the *de novo* motif that is similar to the PLAG1 binding motif MA0163.1 [14] in JASPAR [15]. Source sequence position, source strand, p-value, TFE region, promoter region, TFE and promoter strand, associated gene and TFE position within the gene are shown, as well as the mouse homologs and their developmental function as annotated in the database of transcriptome in mouse early embryos (DBTMEE). The concept TFE, an identifier of transcription start site, was used in our earlier human ZGA study [11].

Sheet MAST Position of sequences similar to the *de novo* PLAG1 motif in the human ZGA TFEs within –2,000 bp upstream to 500 bp downstream from their transcription start site (TSS). The sheet gives the MAST [16] analysis results for the 93 TFEs that contain sites similar to the *de novo* PLAG1 motif. The position of the motif, motif strand, p-value, TFE region, promoter region, TFE and promoter strand, associated gene, and TFE position within the gene are shown.

Sheet AluJSY Position of Alu elements in the human ZGA TFEs within –2,000 bp upstream to 500 bp downstream from their transcription start site (TSS). AluS/J/Y elements were extracted from the RepeatMasker track of the UCSC Genome Browser, and the columns about TFE were joined. The type of element, its position ("left" and "right" within the promoter 0-2,500), TFE region, promoter region, TFE and promoter strand, associated gene, and TFE position within the gene are shown.

File S2. Gene ontology (GO) clusters associated with delayed-activation and delayed-degradation genes
Genes affected by maternal *Plag1* deficiency in 2-cell mouse embryos were assigned to GO categories using topGO library in R [5] using the classic algorithm and Fisher statistics. Top 150 GOs by p-value were then clustered based on their semantic similarity. Clusters 1-8 (delayed up) and 1-6 (delayed down) are shown.

File S3. Gene ontology (GO) clusters associated with up- and downregulated genes during zygotic genome activation (ZGA) in wildtype (WT) mice
Genes up- and downregulated during major ZGA in wild type mouse embryos (2-cell to 8-cell transition) were assigned to GO categories using topGO library in R [5] using the classic

algorithm and Fisher statistics. Top 150 GOs by p-value were then clustered based on their semantic similarity. Clusters 1-9 are shown.

File S4. PLAG1 *de novo* motifs in mouse zygotic genome activation (ZGA) genes
All annotated mouse promoters were scanned for the presence of the *de novo* PLAG1 motif from –2,000 bp upstream to 500 bp downstream of transcriptional start sites (TSSs) using Homer [4]. Delayed-activation genes that have *de novo* PLAG1 motifs in their promoters are shown (symbol, entrez, refSeq) together with the motif sequence, strand and genomic coordinates of the motif as well as the location of the transcription start site (TSS) and the distance relative to the direction of the gene. The last column indicates if the gene is also present among the human ZGA genes [11].

Figure S1. Comparison of human and mouse PLAG1 sequences

```
                                             +-------------------1-+
                     1         2         3         4         5         6
            123456789012345678901234567890123456789012345678901234567890
Human  1    MATVIPGDLSEVRDTQKVPSGKRKRGETKPRKNFPCQLCDKAFNSVEKLKVHSYSHTGER  60
            MATVIPGDLSEVRDTQK PSGKRKRGE+KPRKNFPCQLCDKAFNSVEKLKVHS+SHTGER
Mouse  1    MATVIPGDLSEVRDTQKAPSGKRKRGESKPRKNFPCQLCDKAFNSVEKLKVHSFSHTGER  60

              +--------------------2-+     +-------------------3-+
            6        7         8         9        10        11        12
            123456789012345678901234567890123456789012345678901234567890
Human  61   PYKCIQQDCTKAFVSKYKLQRHMATHSPEKTHKCNYCEKMFHRKDHLKNHLHTHDPNKET  120
            PYKC  QDCTKAFVSKYKLQRHMATHSPEKTHKCNYCEKMFHRKDHLKNHLHTHDPNKET
Mouse  61   PYKCTHQDCTKAFVSKYKLQRHMATHSPEKTHKCNYCEKMFHRKDHLKNHLHTHDPNKET  120

            +-------------------4-+      +-------------------5-+
           12       13        14        15        16        17        18
            123456789012345678901234567890123456789012345678901234567890
Human  121  FKCEECGKNYNTKLGFKRHLALHAATSGDLTCKVCLQTFESTGVLLEHLKSHAGKSSGGV  180
            FKCEECGK+YNTKLGFKRHLALHAATSGDLTCKVCLQ FESTGVLLEHLKSHAGKSSGGV
Mouse  121  FKCEECGKSYNTKLGFKRHLALHAATSGDLTCKVCLQNFESTGVLLEHLKSHAGKSSGGV  180

                +-------------------6-+     +--------------------7-+
           18       19        20        21        22        23        24
            123456789012345678901234567890123456789012345678901234567890
Human  181  KEKKHQCEHCDRRFYTRKDVRRHMVVHTGRKDFLCQYCAQRFGRKDHLTRHMKKSHNQEL  240
            KEKKHQCEHC+RRFYTRKDVRRHMVVHTGRKDFLCQYCAQRFGRKDHLTRHMKKSHNQEL
Mouse  181  KEKKHQCEHCERRFYTRKDVRRHMVVHTGRKDFLCQYCAQRFGRKDHLTRHMKKSHNQEL  240

            123456789012345678901234567890123456789012345678901234567890
Human  241  LKVKTEPVDFLDPFTCNVSVPIKDELLPVMSLPSSELLSKPFTNTLQLNLYNTPFQSMQS  300
            LKVKTEPVDFLDPFTCN+SVPIKDELLPVMSLPSSELLSKPFTNTLQLNLYNTPFQSMQS
Mouse  241  LKVKTEPVDFLDPFTCNMSVPIKDELLPVMSLPSSELLSKPFTNTLQLNLYNTPFQSMQS  300

Human  301  SGSAHQMITTLPLGMTCPIDMDTVHPSHHLSFKYPFSSTSYAISIPEKEQPLKGEIESYL  360
            SGSAHQMITTLPLGMTCPIDMD VHPSHHL+FK PFSSTSYAISIPEKEQPLKGEIESYL
Mouse  301  SGSAHQMITTLPLGMTCPIDMDAVHPSHHLAFKCPFSSTSYAISIPEKEQPLKGEIESYL  360

Human  361  MELQGGVPsssqdsqassssKLGLDPQIGSLDDGAGDlslskssisisDPLNTPALDFSQ  420
            MELQGG P SS     +SSSKLGL+PQ GS DDGAGDLSLSKSSISISDPL+TPALDFSQ
Mouse  361  MELQGGAP-SSSQDSPASSSKLGLEPQSGSPDDGAGDLSLSKSSISISDPLSTPALDFSQ  419

Human  421  LFNFIPLNGPPYNPLSVGSLGMSYSQEEAHSSVSqlppqtqdlqdpANTIGLGslhslsa  480
            LFNFIPLNGPPYNPLSVGSLGMSYSQEEAHSSVSQLP QTQDLQDPANT+GL SLHSLSA
Mouse  420  LFNFIPLNGPPYNPLSVGSLGMSYSQEEAHSSVSQLPTQTQDLQDPANTVGLSSLHSLSA  479

Human  481  aftsslststtlPRFHQAFQ  500
            AFTSSLS+STTLPRFHQAFQ
Mouse  480  AFTSSLSSSTTLPRFHQAFQ  499
```

Comparison of amino acid sequences between human PLAG1 (RefSeq NP_002646.2) and mouse PLAG1 (NP_064353.2) by NCBI blastp [18] indicating 94% similarity. The seven C2H2 zinc-finger domains are labeled (+---+) based on UniProt protein knowledge base[19] (entry Q6DJT9). Domains 6 and 7 bind to the "core" of the PLAG1 consensus sequence while domain 3 binds to the "cluster" [Figure 1c *(v)* and [20]]. The amino acid sequences of these three domains are identical between mice and humans except for position 191 (red) within domain 6. However, human PLAGL1 has a glutamic acid residue (E) at the corresponding position of the PLAG1 D191E, and binding preference to G-rich "core" was highly conserved in both of PLAG1 and PLAGL1[21]. Moreover, similarity of the C2H2 domains of mousePLAG1 to human PLAG1 is higher than human PLAGL1. Therefore, although mouse PLAG1 has one inconsistent residue within the C2H2 domain for binding, preference of the binding site sequences would be identical between human and mouse PLAG1

Figure S2. *Plag1* deficiency does not have significant effects on ovaries and uterus.

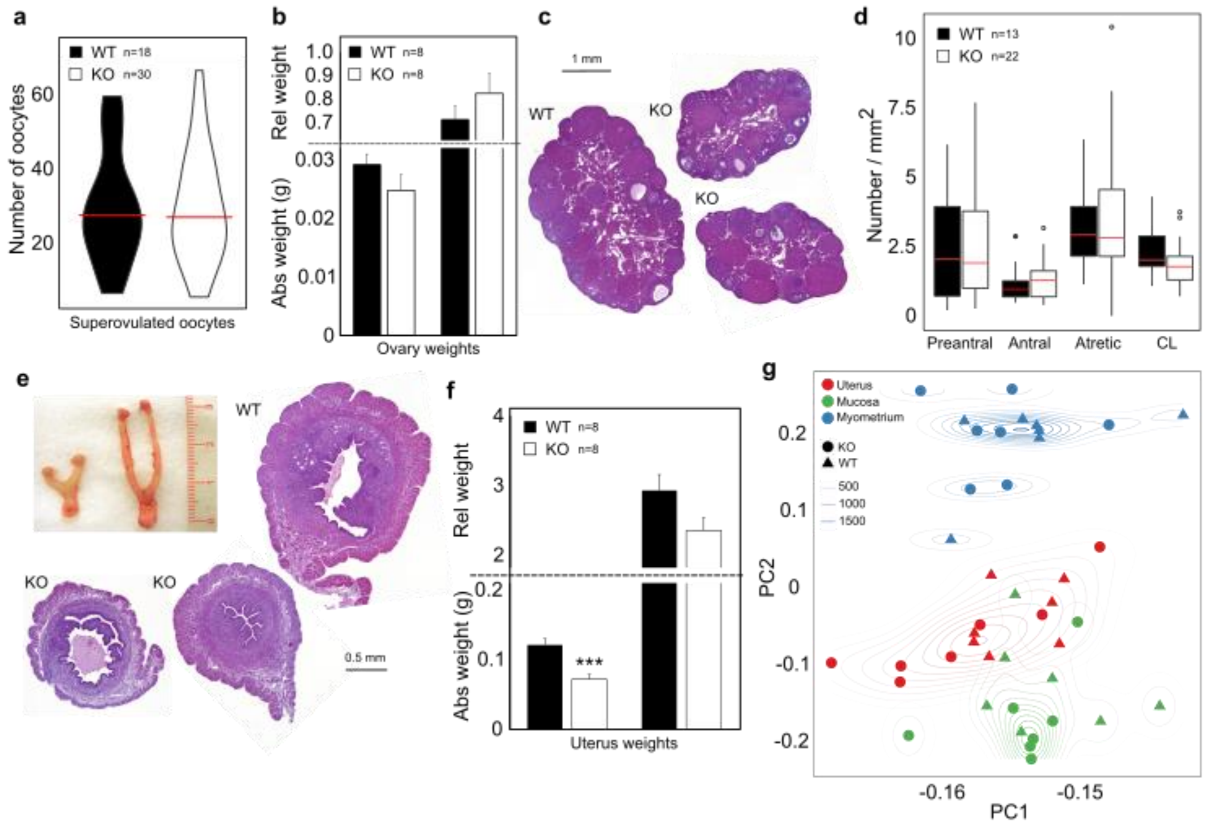

(a) Number of oocytes collected after superovulation presented as violin plots. The median is marked as a red line. **(b)** Absolute and relative (body weight-adjusted) weights of ovaries, shown as means + SEM. **(c)** Representative images of hematoxylin-eosin-stained ovarian cross sections from wildtype (WT) and *Plag1* knockout (KO) mice. The scale bar is 1 mm. **(d)** Numbers of preantral, antral and atretic follicles as well as corpora lutea (CL) per mm^2 of ovary. Data are presented as median (red line), interquartile ranges (box), and non-outlier ranges (whisker). Outliers are depicted as dots. **(e)** Representative photos and hematoxylin-eosin-stained histological images of WT and *Plag1* KO uteri. The scale bar is 500 µm. **(f)** Absolute and relative (body weight-adjusted) uterine weights presented as means + SEM. **(g)** Principal component (PC) analysis of global gene expression in WT and KO uterus samples (N = 7) divided into “uterus” (a piece of uterine horn), “mucosa” (cells scraped from the endometrial surface of the uterine horn), and “myometrium” (tissue left after mucosa has been removed).
Statistical analysis by *t*-test (a, b, f) or two-way ANOVA (d); ***p < 0.001.

Figure S3. Size of the mRNA and spike-in reference RNA libraries

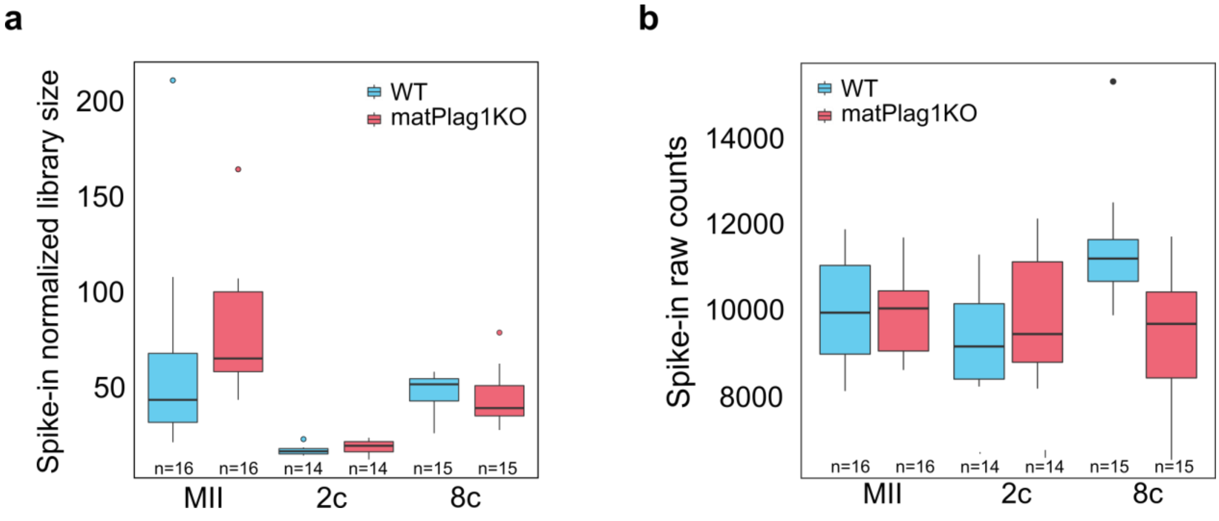

The size of the **(a)** spike-in normalized mRNA libraries and **(b)** spike-in reference RNA libraries recovered through RNA-sequencing. The size did not significantly vary between genotypes. MII, MII oocyte; 2c, 2-cell stage embryo; 8c, 8-cell stage embryo.

Figure S4. Expression of *PLAG1* in human embryos

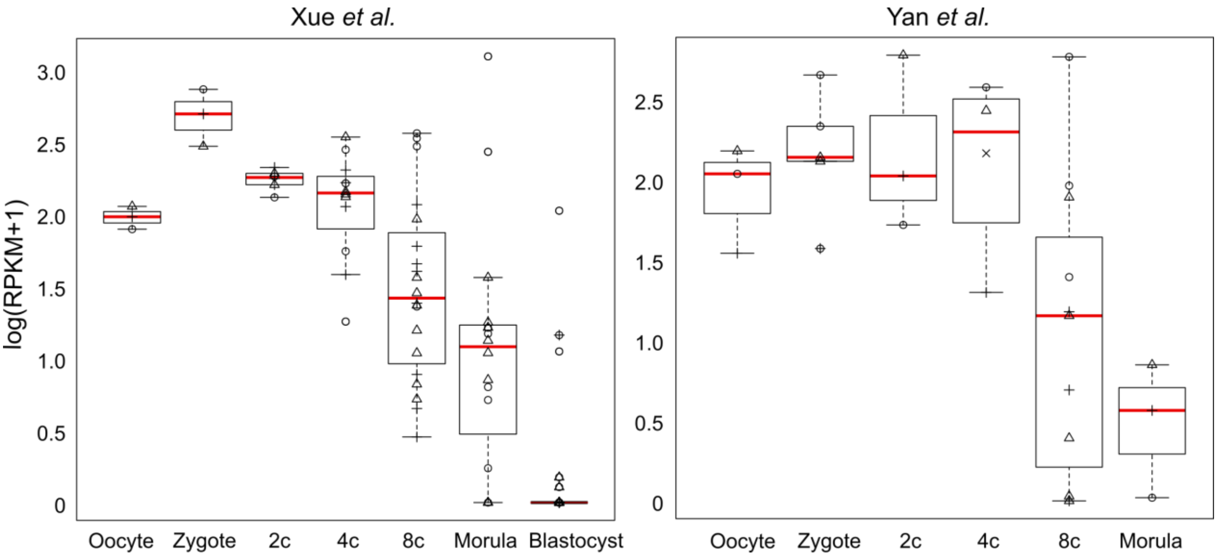

PLAG1 expression in human pre-implantation embryos in two independent single-cell RNA-sequencing datasets. The normalized expression matrices were downloaded from GEO with the accession numbers GSE44183 [22] and GSE36552 [23]. Expression is shown as log-transformed RPKM values by developmental stage. A constant (1) was added to the RPKM values prior to log-transformation to avoid negative values. The data are presented as box plots where median is shown as a red line, edges of the box are the upper and lower quartiles, and the whiskers show the highest and lowest values while excluding outliers. Cells from the same embryo in each developmental stage are depicted with the same shape. 2c, 2-cell; 4c, 4-cell; 8c, 8-cell; RPKM, reads per kilobase million.

Figure S5. Expression of *Plag1* transcripts in mouse ovary as shown by X-gal staining

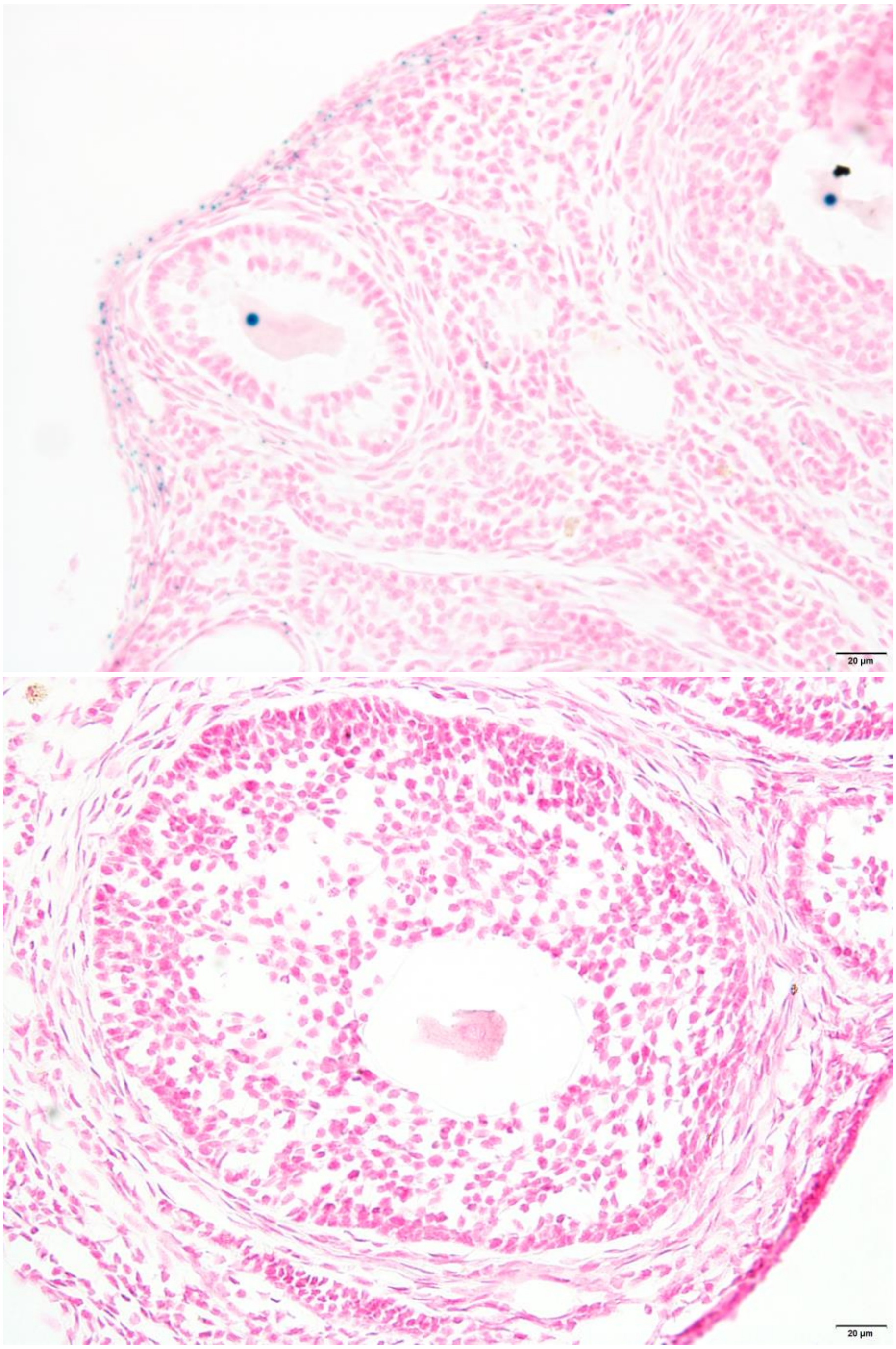

The mutant allele in the *Plag1KO* animals consists of the *lacZ* gene that replaces the entire open reading frame of *Plag1*, enabling visualization of *Plag1* promoter activity using X-gal staining. The top picture displays *lacZ*-positive cells (blue) in a representative ovary section of a *Plag1KO* mouse, aged between 4-5 months, as determined by X-gal staining. Expression of *lacZ* was found mostly in the nuclei of growing oocytes and in the tunica albugina. The bottom picture shows a negative control for X-gal staining (WT mouse), showing no blue signal. The scale bars are 20 μm.

Figure S6. Gene set comparison of mouse and human zygotic genome activation (ZGA) genes

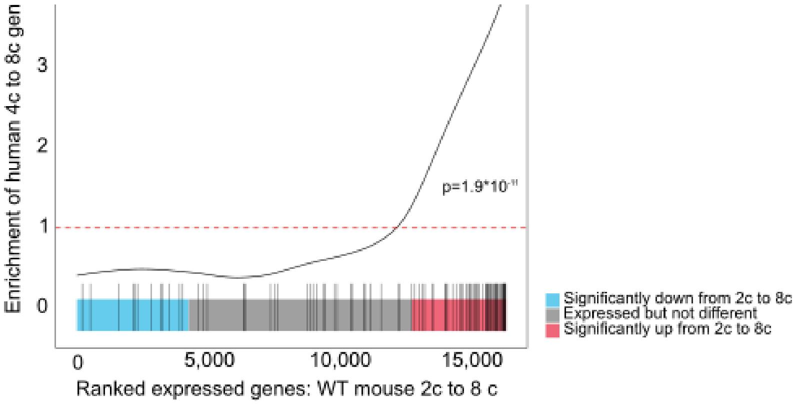

Gene set enrichment analysis comparing gene expression changes during mouse and human ZGA. Mouse ZGA genes are displayed on the x-axis ranked by the level of expression change, and human ZGA gene that have a mouse orthologue are depicted with black vertical lines. Enrichment of the human genes among the mouse genes is depicted wth the black line. No enrichment level (=1) is shown as red dotted line. Significance of the enrichment was tested with the GeneSet test function.

Figure S7 Frequency of *de novo* PLAG1 motifs and B1 elements in delayed-activation and delayed-degradation

a

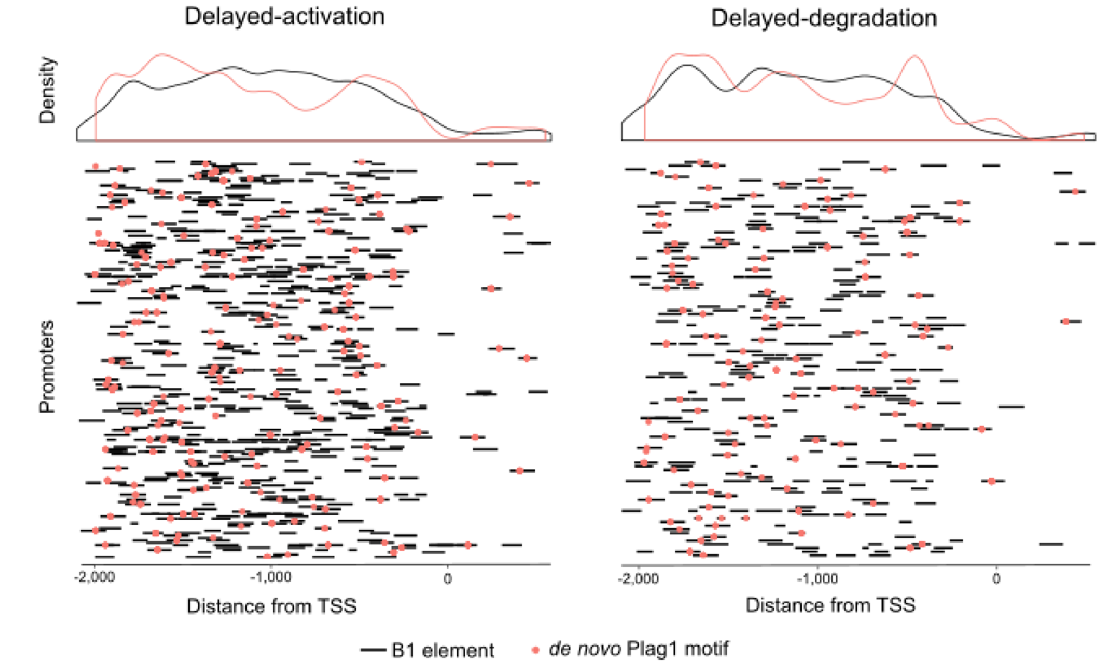

b

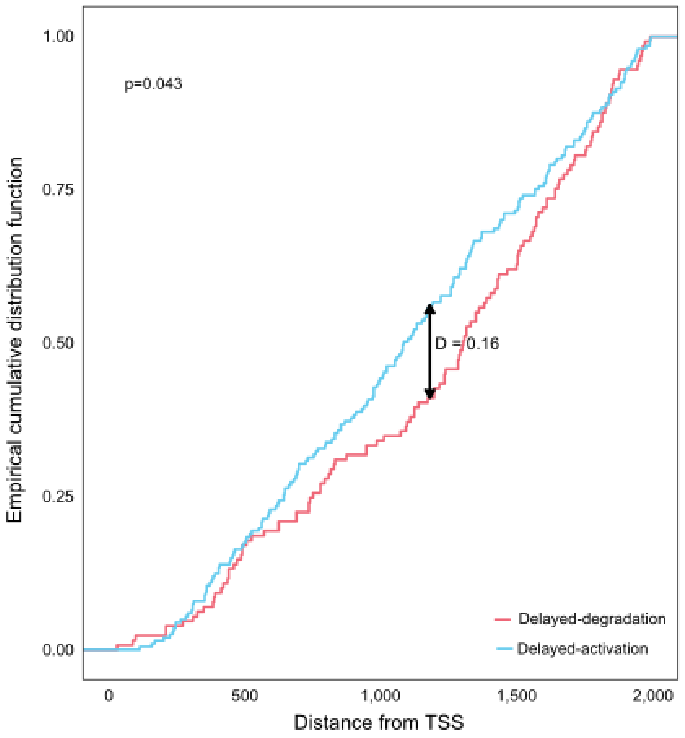

(a) Location of B1 repetitive elements (black lines) and *de novo* PLAG1 motifs (red dots) along the promoters of delayed-activation and delayed-degradation genes from -2,000 bp to +500 bp around the transcription start site (TSS). Enrichment of the sites as shown as color-coded lines above the graphs.
(b) Empirical cumulative distribution function showing the distribution of *de novo* PLAG1 motifs along the promoters of delayed-activation and delayed-degradation genes.

References

1 Juma, A. R. *et al.* PLAG1 deficiency impairs spermatogenesis and sperm motility in mice. *Sci Rep* **7**, 5317, doi:10.1038/s41598-017-05676-4 (2017).

2 Krjutskov, K. *et al.* Single-cell transcriptome analysis of endometrial tissue. *Hum Reprod* **31**, 844-853, doi:10.1093/humrep/dew008 (2016).

3 Kim, D. *et al.* TopHat2: accurate alignment of transcriptomes in the presence of insertions, deletions and gene fusions. *Genome Biol* **14**, R36, doi:10.1186/gb-2013-14-4-r36 (2013).

4 Heinz, S. *et al.* Simple combinations of lineage-determining transcription factors prime cis-regulatory elements required for macrophage and B cell identities. *Mol Cell* **38**, 576-589, doi:10.1016/j.molcel.2010.05.004 (2010).

5 R: A language and environment for statistical computing (R Foundation for Statistical Computing, Vienna, Austria, 2010).

6 Robinson, M. D., McCarthy, D. J. & Smyth, G. K. edgeR: a Bioconductor package for differential expression analysis of digital gene expression data. *Bioinformatics* **26**, 139-140, doi:10.1093/bioinformatics/btp616 (2010).

7 Gu, Z., Eils, R. & Schlesner, M. Complex heatmaps reveal patterns and correlations in multidimensional genomic data. *Bioinformatics* **32**, 2847-2849, doi:10.1093/bioinformatics/btw313 (2016).

8 Trapnell, C. *et al.* The dynamics and regulators of cell fate decisions are revealed by pseudotemporal ordering of single cells. *Nat Biotechnol* **32**, 381-386, doi:10.1038/nbt.2859 (2014).

9 Yu, G. *et al.* GOSemSim: an R package for measuring semantic similarity among GO terms and gene products. *Bioinformatics* **26**, 976-978, doi:10.1093/bioinformatics/btq064 (2010).

10 Ritchie, M. E. *et al.* limma powers differential expression analyses for RNA-sequencing and microarray studies. *Nucleic Acids Res* **43**, e47, doi:10.1093/nar/gkv007 (2015).

11 Tohonen, V. *et al.* Novel PRD-like homeodomain transcription factors and retrotransposon elements in early human development. *Nat Commun* **6**, 8207, doi:10.1038/ncomms9207 (2015).

12 Gupta, S., Stamatoyannopoulos, J. A., Bailey, T. L. & Noble, W. S. Quantifying similarity between motifs. *Genome Biol* **8**, R24, doi:10.1186/gb-2007-8-2-r24 (2007).

13 Bailey, T. L. & Elkan, C. Fitting a mixture model by expectation maximization to discover motifs in biopolymers. *Proc Int Conf Intell Syst Mol Biol* **2**, 28-36 (1994).

14 Meng, X., Brodsky, M. H. & Wolfe, S. A. A bacterial one-hybrid system for determining the DNA-binding specificity of transcription factors. *Nat Biotechnol* **23**, 988-994, doi:10.1038/nbt1120 (2005).

15 Sandelin, A., Alkema, W., Engstrom, P., Wasserman, W. W. & Lenhard, B. JASPAR: an open-access database for eukaryotic transcription factor binding profiles. *Nucleic Acids Res* **32**, D91-94, doi:10.1093/nar/gkh012 (2004).

16 Bailey, T. L. & Gribskov, M. Combining evidence using p-values: application to sequence homology searches. *Bioinformatics* **14**, 48-54 (1998).

17 Hubley, R. *et al.* The Dfam database of repetitive DNA families. *Nucleic Acids Res* **44**, D81-89, doi:10.1093/nar/gkv1272 (2016).

18 Altschul, S. F., Gish, W., Miller, W., Myers, E. W. & Lipman, D. J. Basic local alignment search tool. *J Mol Biol* **215**, 403-410, doi:10.1016/S0022-2836(05)80360-2 (1990).

19 Pundir, S., Martin, M. J. & O'Donovan, C. UniProt Protein Knowledgebase. *Methods Mol Biol* **1558**, 41-55, doi:10.1007/978-1-4939-6783-4_2 (2017).

20 Voz, M. L., Agten, N. S., Van de Ven, W. J. & Kas, K. PLAG1, the main translocation target in pleomorphic adenoma of the salivary glands, is a positive regulator of IGF-II. *Cancer Res* **60**, 106-113 (2000).

21 Hensen, K., Van Valckenborgh, I. C., Kas, K., Van de Ven, W. J. & Voz, M. L. The tumorigenic diversity of the three PLAG family members is associated with different DNA binding capacities. *Cancer Res* **62**, 1510-1517 (2002).

22 Xue, Z. *et al.* Genetic programs in human and mouse early embryos revealed by single-cell RNA sequencing. *Nature* **500**, 593-597, doi:10.1038/nature12364 (2013).
23 Yan, L. *et al.* Single-cell RNA-Seq profiling of human preimplantation embryos and embryonic stem cells. *Nat Struct Mol Biol* **20**, 1131-1139, doi:10.1038/nsmb.2660 (2013).
